# Southern Medical and Surgical Journal

**Published:** 1850-11

**Authors:** 


### SOUTHERN MEDICAL AND SURGICAL JOURNAL.

Owing to a temporary derangement in the printing office of the Medical Examiner, by which we were prevented from seeing all the proofs, we failed to give credit to our esteemed cotemporary, for the excellent article on the Renal Circulation by M. Bernard, communicated to the Southern Medical and Surgical Journal, by Dr. Harris, and which appeared in our Record of Medical Science in the September number. It was entirely accidental.

We wish that those who copy from the "Examiner" would also "render unto Cæsar the things that are Cæsar's."

